# Perceived Impact of Gambling Advertising can Predict Gambling Severity among Patients with Gambling Disorder

**DOI:** 10.1007/s10899-024-10342-2

**Published:** 2024-07-30

**Authors:** Hibai Lopez-Gonzalez, Roser Granero, Fernando Fernández-Aranda, Mark D. Griffiths, Susana Jiménez-Murcia

**Affiliations:** 1 Department of Library, Information Science and Audiovisual Communication, DHIGECS-COM, CRICC, University of Barcelona, Melcior de Palau 140, 08014 Barcelona, Spain; 2 Department of Psychobiology and Methodology, Autonomous University of Barcelona, Barcelona, 08193 Spain; 3 Psychoneurobiology of Eating and Addictive Behaviors Group, Neurosciences Programme, Bellvitge Biomedical Research Institute (IDIBELL), Barcelona, 08908 Spain; 4 Ciber Physiopathology of Obesity and Nutrition (CIBERObn), Instituto de Salud Carlos III, Barcelona, 08908 Spain; 5 Clinical Psychology Unit, Bellvitge University Hospital- Bellvitge Biomedical Research Institute (IDIBELL), Barcelona, 08907 Spain; 6 Department of Clinical Sciences, School of Medicine, University of Barcelona, Barcelona, 08907 Spain; 7 International Research Gambling Unit, Psychology Department, Nottingham Trent University, Nottingham, UK

**Keywords:** Gambling, Gambling advertising, Gambling marketing, Gambling preference, Online gambling, Gambling regulation

## Abstract

There is growing evidence that gambling advertising disproportionately affects those experiencing more severe gambling harm. Such association has been studied by recruiting gamblers using online panels, by analysing registered users’ data from gambling websites, and through surveys and focus group interviews. However, it is thought that these methods tend to overestimate gambling severity. The present study employed a sample of gamblers with a verified gambling disorder diagnosis (*N* = 210, 7.1% females, M_age_ = 39.4 years) recruited for a period of under two years at a large public hospital. It examined the relationship between self-reported impact of gambling advertising, gambling preference (strategic versus non-strategic) and gambling modality (online versus in-person). The results indicated that higher perceived impact of gambling advertising predicted higher gambling severity, which supports previous findings obtained from non-clinical settings. However, contrary to what was expected, strategic gambling and online gambling were not associated with higher perceived impact of gambling advertising, even though these groups are believed to be exposed to more gambling marketing and advertising from gambling operators. The study aligns well with available scientific evidence proposing further restrictions on gambling advertising regulation due to their disproportionate impact on those already experiencing gambling harm.

**Supplementary Information:**

The online version contains supplementary material available at 10.1007/s10899-024-10342-2.

## Introduction

Gambling providers rely on marketing and advertising strategies to persuade consumers to engage with products that in the long run will lose them money (Dow Schüll, 2012; Hing et al., 2014). To counterbalance the negative financial expectation of gambling products (Levitt, 2004) – which in theory should make gambling unattractive for consumers – marketing and advertising communications emphasize some aspects of gambling while deemphasizing others in an effort to present it in a more favourable light. Commercial messages include mentions to the degree of control that gamblers have over their gambling platforms and sites (i.e., illusion of control), present gamblers as glamorous people with analytical skills, create an imaginary of big wins as dreams that consumers should feel entitled to, and in general, reinforce the idea that being successful in gambling is more likely than it actually is (Binde, 2007; Lopez-Gonzalez et al., 2018; McMullan & Miller, 2008; Sklar & Derevensky, 2011).

Such communications tactics might be perceived as harmful by gamblers, especially by those in vulnerable situations, affecting the discontinuation of gambling and/or their recovery from gambling problems. Therefore, the present study explored how gamblers with gambling disorder (GD) perceive the impact of gambling advertising. The study departs from previous gambling advertising investigations in that it uses a sample of clinically diagnosed individuals undergoing treatment for GD, something only done once before, to the best of our knowledge, in a sample of U.S.-based gamblers with GD within a study in which gambling advertising was not the main focus (Grant & Kim, 2001). In our study, the robust diagnostic protocol conducted by professional psychologists working in a hospital facility to assess GD makes the sample composition especially significant. This is because traditionally gambling advertising samples are recruited via online panels, which show a tendency to overestimate the number of gambling problems, as indicated by the fact that when these same individuals are assessed by means of in-person interviews, the cases of GD decline as well as their overall gambling severity scores (Sturgis & Kuha, 2022). The following section reviews the relevant empirical evidence and outlines the main hypotheses of the study.

### Literature Review and Hypothesis Development

The last decade has seen a proliferation of gambling stimuli in the form of various marketing and advertising techniques including television and radio adverts, banners on websites and pop ups, and social media promotions (Gainsbury et al., 2016). A large proportion of the growth has been due to online sports betting advertising. In Australia, a person watching a sport broadcast was estimated to be exposed on average to 106 episodes of gambling marketing per game (Lindsay et al., 2013). In another Australian study, children aged 5 to 12 years had to associate magnets from sport teams with risk products (alcohol, junk food, and gambling). They found that sports teams were often associated with gambling logos, even then these gambling firms did not sponsor the team (Bestman et al., 2015). Consequently, there appears to be a growing consensus that an uncontrolled proliferation of gambling advertising is incompatible with public health objectives, and that gambling advertising must be regulated more tightly than it has been to date (Browne et al., 2019; Hing et al., 2017; Kim et al., 2013; Newall, 2018; Pitt et al., 2016; Sharman, 2022; Sproston et al., 2015).

The mounting pressure that gambling regulators are imposing on what gambling advertisers can do is incentivising a transformation in the gambling advertising market. Traditional television and radio advertisements are being substituted by social media marketing strategies in which limiting the exposure of vulnerable groups becomes harder (Guillou-Landreat et al., 2021). A mixed methods study using big data from *Twitter* (now *X*) and a manual codification technique found that gambling advertisements on social media were often unclear and inexplicit about their promotional nature, and that the normalisation effect they produce can be larger than in traditional advertising because of the sheer volume of inducements some users are exposed to (Rossi et al., 2021).

Whether (and how) gambling advertising affects gamblers is a matter of dispute. Binde (2014) estimated that gambling advertising plays a secondary role in the development of gambling disorder (GD) when compared to other factors such as personality traits, biological characteristics, product availability and other structural characteristics including the speed of play and the duration of the games (Parke & Griffiths, 2007). Theorising and demonstrating a mechanism of gambling influence is difficult for many reasons, among which rank high the long-term effects of advertising. Some studies have tried to examine the short-term effects of gambling enticements by showing sports bettors gambling stimuli from sports television coverage (Lamont et al., 2016). However, assessing long-term effects often implies the interference of confounding variables that make much harder to isolate gambling advertising as the predictor variable. In a critical and meta-analytic review of the published evidence concerning the gambling advertising effects on gambling attitudes, intentions, and behaviours, the authors reported a positive association, but highlighted the absence of longitudinal and experimental research, especially if compared to the analogous areas of tobacco and alcohol advertising research (Bouguettaya et al., 2020).

The evidence suggesting an association between gambling severity and gambling advertising is mounting (McGrane et al., 2023). Several studies from Australia have found associations between higher problem gambling severity and exposure to gambling promotions among adults (Hing et al., 2015), university students (Hing et al., 2013), internet sports bettors (Hing et al., 2017), and secondary school students (Noble et al., 2022). Similar results have been obtained in Quebec, Canada, from a sample of adolescents in which those reporting higher exposure and recall of gambling advertising also reported higher scores for gambling problems (Derevensky et al., 2010a). Furthermore, two studies from Norway also found an association between gambling severity and exposure to gambling advertising (Hanss et al., 2015; Syvertsen et al., 2022). Additional quantitative studies reporting a relationship between gambling advertising exposure and gambling problems include Clemens et al. (2017) and Gavriel Fried et al. (2010). Qualitative approaches to understanding the influence of gambling advertising have also reported an association with gambling harm. Gambling advertisements cause distress to people undergoing treatment for GD because they find it difficult to resist them (Deans et al., 2017; Lopez-Gonzalez et al., 2020a; Thomas et al., 2012). Therefore, the present study hypothesised that: *higher perceived impact of gambling advertising will be associated with higher scores on gambling severity* (H_1_).

An important question to ask is whether the relationship between gambling severity and gambling advertising equally affects gamblers from all gambling preferences and modalities. Several previous studies have explored the behavioural, biological, and personality differences between strategic gamblers (e.g., those playing poker, sports betting) and non-strategic gamblers (e.g., those playing slot machines, lottery, bingo, and roulette). In comparison, strategic gamblers are more likely to be male, younger, sensation seekers, impulsive, with higher education, higher economic level, and with earlier GD onset and greater severity than non-strategic gamblers (Grant et al., 2012; Jiménez-Murcia et al., 2020). Moreover, strategic gamblers tend to be more analytical while non-strategic gamblers resort to intuition more often (Mouneyrac et al., 2018).

It could be argued that strategic gamblers are more likely to be targeted by gambling advertising and, therefore, perceive a greater impact from it. First, sports betting and poker have been far more advertised in the past decade, becoming a ubiquitous presence in all sorts of sporting competitions (Browne et al., 2019; Lopez-Gonzalez & Griffiths, 2018; Newall et al., 2019; Sharman et al., 2019). The sudden proliferation of sports betting advertising including the endorsement of betting brands by high profile athletes – who are seen as icons by many children and adolescents – has arguably been an essential factor in expediting regulatory changes in gambling advertising (Hörnle & Carran, 2018; Lopez-Gonzalez & Griffiths, 2016). Second, data from paid-for gambling adverts in the UK showed greater expenditure on poker, sports betting, and online casino (which include some types of non-strategic gambling) than on other forms of gambling (Critchlow et al., 2022). Third, being of younger age might make strategic gamblers not only more likely to be exposed to gambling stimuli but also to be more easily persuaded by their communication tactics. In fact, in connection with their overall higher scores on impulsivity and sensation seeking, strategic gamblers may constitute a more vulnerable group to the harms of gambling advertising than non-strategic gamblers. Based on this rationale, the present study hypothesised that: *higher perceived impact of gambling advertising will be higher for gamblers engaging in strategic forms of gambling than non-strategic forms of gambling* (H_2_). Finally, strategic gamblers are more likely to gamble online because their preferred gambling forms are more often online than it is for non-strategic gamblers. Also, the younger age of strategic gamblers makes them more likely to consume online as opposed to in-person gambling. Therefore. The present study hypothesised that: *higher perceived impact of gambling advertising will be higher for gamblers engaging in online rather than in-person gambling* (H_3_).

## Materials and methods

### Participants and Procedure

The study recruited a sample of consecutive treatment-seeking adults diagnosed with GD (*N* = 210). They were recruited from June 2019 until January 2021 in the Behavioural Addictions Unit of a public hospital in the greater area of Barcelona, which covers a populated area of over two million inhabitants. GD was diagnosed by two means. First, individuals were assessed using both DSM-5 criteria (American Psychiatric Association, 2013) and SOGS criteria (Lesieur & Blume, 1987) for gambling disorder by completing a self-administered questionnaire. Second, an in-person interview with a clinical psychologist confirmed the diagnosis. Individuals were eligible to participate if they met the following criteria: (i) being an adult, (ii) having a diagnosis of GD, (iii) signing the content form, (iv) and GD being their primary reason to seek for help. There were no specific exclusion criteria.

The participants completed a paper-and-pencil self-administered questionnaire before beginning their cognitive-behavioural therapy. From the 218 questionnaires completed, eight were discarded (because two answered every single item with the same score, one did not consent to participate, and five did not provide some necessary personal details). This resulted in a final sample of 210 participants.

### Measures

*Impacts of Gambling Advertising Scale* (Hanss et al., 2015). The IGAS comprises nine items partially derived from a previous scale (Derevensky et al., 2010b). More specifically, the scale consists of three subscales designed to assess the perceived impact of gambling advertising: knowledge (e.g., *“I am more likely to gamble after seeing a gambling advertisement”*), awareness (e.g., *“I don’t pay attention to gambling advertisements”*), and involvement (e.g., *“Gambling advertisement has increased my knowledge of gambling providers”*). Using a translation and back-translation technique, a Spanish version of the scale was developed. For the Spanish version, participants rated the nine items on a four-point scale (from 1 = *“strongly agree”* to 4 = *“strongly disagree”*), meaning that lower scores indicated a greater perceived impact of gambling advertising. In the present sample, internal consistency was very good (α = 0.86).

*Consumer Sentiment Toward Marketing* (Gaski & Etzel, 1986). This instrument comprises a number of subscales. The present study only used the seven-item “Advertising for Products” subscale which assesses attitudes towards advertising. The items are rated on a five-point scale (1 *= “Agree strongly”*, 5 = *“Disagree strongly”*). The Spanish adaptation of the subscale included a modification to represent gambling advertising instead of advertising more generally. The modification was carried out by adding the word “gambling” before the word “advertising” in six of the items, and before the word “ads” in the remaining item. In the present study, the internal consistency was very good α = 0.80.

*Consumer Self-Confidence on Persuasion Knowledge* (Bearden et al., 2001). The six-item CSCPK assesses how confident participants are about their degree of knowledge in identifying marketing strategies. Items are rated from 1 (*“extremely uncharacteristic”*) to 5 (*“extremely characteristic”*) concerning their perceived knowledge of persuasion strategies (e.g., *“I know when an offer is too good to be true”*). The scale was adapted into Spanish for the present study and had excellent internal consistency (α = 0.91).

*South Oaks Gambling Screen* (Lesieur & Blume, 1987)The SOGS is a 20-item self-administered screening tool that assesses gambling problems. Individuals are categorized into three groups based on how they score: non-problem, probable pathological, and problem gamblers. In the present study, the internal consistency was good (α = 0.75).

*Sociodemographic and other gambling-related variables*. Information about a number of sociodemographic was collected for the study including age, gender, marital status, employment status, educational attainment, and social index (Hollingshead, 2011). Particularly relevant for the present study, gambling forms (strategic, non-strategic, and mixed), and gambling modality (in-person, online, and mixed) were asked about. Moreover, information concerning onset and duration of GD, engaging in illegal acts to fund gambling behaviour, and indebtedness was also collected.

### Ethics

The research was planned and executed in accordance with the Declaration of Helsinki of 1975, revised in 2000, and it was approved by the Ethics Committee of Bellvitge University Hospital (Ref: PR338/17-CSI 18/04). All participants provided informed consent and received no monetary compensation for participating.

### Statistical Analysis

Stata18 for Windows (StataCorp, 2023) was used for the statistical analysis. The means obtained in the scale scores assessing the impact of gambling advertising were compared between the groups (defined by sex and the gambling activity profile) through analysis of covariance (ANCOVA). These models were adjusted by sex, age and other gambling comorbid activities (depending on the concrete comparisons). In these analyses, Cohen’s *d* coefficients indicated the effect size for the pairwise comparisons, and it was considered null magnitude for |*d*|<0.20, low-poor for |*d*|>0.20, moderate-medium for |*d*|>0.50 and large-high for |*d*|>0.80) (Cohen, 1988). Additionally, Finner’s method was used to control increase in Type-I error due to the multiple significance tests (Finner & Roters, 2001).

The association between perceived impact of gambling advertising and the clinical profile analysed in the study (gambling related measures) was calculated through partial correlations (also adjusted by sex and age). Due to the strong association between statistical significance in correlation model and sample size (i.e., low coefficients tend to achieve significant results with large samples, while high coefficients tend to non-significance with low samples), the present study interpreted relevant correlations’ coefficients within the mild-moderate (|*R*|>0.24) to high-large range (|*R*|>0.37) (Kelley & Preacher, 2012).

Poisson regression assessed the predictive capacity of the (i) sociodemographic profile, (ii) clinical variables, and (iii) perceived impact of the gambling advertising on the severity of the clinical profile on the GD symptom level (defined as the SOGS total score).

## Results

### Sample Description

Most participants were males (92.9%), single (53.8%), of primary education level (45.2%), and reported mean-low to low socio-economic positions (77.2%). The mean age was 39.4 years (SD = 13.3). Regarding the gambling profile, the most preferred forms of gambling were non-strategic (38.6%), with the majority gambling in-person (72.9%). The mean age of onset of gambling-related problems was 28.9 years (SD = 11.9) and the mean duration of the problems was 5.2 years (SD = 5.7). One-fifth of the participants (19.5%) reported the presence of debts related with their gambling activity, and a quarter also indicated illegal acts due to the GD (26.2%). Table S1 (supplementary material) contains the description for all the variables analysed in the study.

### Gambling-Related Variables Associated to the Impact of Gambling Advertising

Tables 1 and 2 show the results of the ANCOVA comparing the mean scores in the impact of gambling advertising scales. No significant association was found between the factor scores with sex and age (Table 1), nor with the gambling preferences (Table 2). Gambling modality (in-person versus online) was neither associated with gambling severity.

**Table 1:** Association of perceived impact of gambling advertisement with sex and age (*N*=210)

	^1^Women (*n*=15)		^1^Men (*n*=195)				^2^Age (*n*=210)
	Mean	SD	Mean	SD	*p*	|d|	|*R*|
IGAS: Involvement	2.59	0.93	2.87	0.96	0.282	0.29	0.096
IGAS: Awareness	2.53	0.86	2.60	0.94	0.771	0.08	0.120
IGAS: Knowledge	2.15	1.10	2.64	1.09	0.092	0.45	0.050
IGAS: Total	2.48	0.73	2.76	0.81	0.195	0.36	0.110
Bearden et al.: Total	3.07	1.19	2.99	1.18	0.789	0.07	-0.040
Gaski and Etzel: Total	1.50	0.45	1.70	0.68	0.261	0.35	0.017

Note.^1^ANCOVA adjusted by age. ^2^Partial correlation adjusted by sex. SD: standard deviation

**Table 2:** Association of perceived impact of gambling advertisement with gambling preferences (*N*=210)

Gambling preference ^®^	Non-strategic (*n*=81)		Strategic (*n*=76)		Mixed (*n*=53)		Non-strategic vs. strategic		Non-strategic vs. mixed		Strategic vs. mixed	
	Mean	SD	Mean	SD	Mean	SD	*p*	|d|	*p*	|d|	*p*	|d|
IGAS: Involvement	2.81	0.95	2.94	0.94	2.78	1.00	0.444	0.15	0.907	0.02	0.372	0.16
IGAS.: Awareness	2.67	0.94	2.58	0.91	2.50	0.97	0.615	0.10	0.336	0.17	0.664	0.08
IGAS: Knowledge	2.74	1.10	2.51	1.10	2.55	1.09	0.287	0.20	0.352	0.17	0.866	0.03
IGAS: Total	2.76	0.76	2.77	0.83	2.67	0.83	0.957	0.01	0.544	0.11	0.515	0.12
Bearden et al.: Total	2.88	1.10	3.16	1.20	2.91	1.25	0.213	0.24	0.890	0.03	0.259	0.20
Gaski and Etzel: Total	1.73	0.59	1.66	0.72	1.67	0.73	0.623	0.10	0.635	0.09	0.973	0.01
*Gambling modality* ^®^	*In-person* (*n*=153)		*Online* (*n*=44)		*Mixed* (*n*=13)		*In-person* *vs. online*		*In-person* *vs. mixed*		*Online* *vs. mixed*	
	*Mean*	*SD*	*Mean*	*SD*	*p*	*|d|*	*p*	*|d|*	*p*	*|d|*	*p*	*|d|*
IGAS: Involvement	2.85	0.96	2.83	0.88	2.96	1.14	0.916	0.02	0.693	0.10	0.676	0.12
IGAS: Awareness	2.60	0.92	2.49	0.90	2.85	1.20	0.478	0.13	0.367	0.23	0.224	0.34
IGAS: Knowledge	2.67	1.10	2.41	1.02	2.50	1.29	0.178	0.25	0.588	0.14	0.793	0.08
IGAS: Total	2.76	0.79	2.66	0.80	2.83	1.02	0.503	0.12	0.742	0.08	0.500	0.19
Bearden et al.: Total	2.93	1.13	3.25	1.27	2.87	1.35	0.126	0.27	0.864	0.05	0.311	0.29
Gaski and Etzel: Total	1.72	0.66	1.61	0.66	1.64	0.81	0.357	0.17	0.680	0.11	0.889	0.04

Note^1^ANCOVA adjusted by sex and age. SD: standard deviation

The ANCOVA (adjusted by sex and age) further compared the mean scores achieved in the impact of gambling advertising scales between participants with different gambling activities (sports betting and poker) within the strategic gambling preference. While no statistical difference was found comparing those who reported engaging in sports betting (versus those who did not), those with poker-related problems reported a lower mean score on the IGAS Involvement subscale and the IGAS total score Table 3.

**Table 3:** Association of perceived impact of gambling advertisement with gambling type

	^1^Sports betting						^2^Poker					
	No (*n*=146)		Yes (*n*=64)				No (*n*=195)		Yes (*n*=15)			
	*Mean*	*SD*	*Mean*	*SD*	*p*	*|d|*	*Mean*	*SD*	*Mean*	*SD*	*p*	*|d|*
IGAS: Involvement	2.877	0.94	2.788	0.99	0.618	0.09	2.889	0.95	2.342	0.86	0.034*	**0.60** ^**†**^
IGAS: Awareness	2.585	0.94	2.613	0.92	0.872	0.03	2.608	0.94	2.407	0.89	0.426	0.22
IGAS: Knowledge	2.66	1.13	2.492	1.01	0.414	0.16	2.637	1.09	2.236	1.08	0.174	0.37
IGAS: Total	2.764	0.78	2.684	0.85	0.592	0.10	2.771	0.80	2.334	0.79	0.044*	**0.55** ^**†**^
Bearden: Total	3.075	1.13	2.806	1.28	0.222	0.22	3.024	1.16	2.592	1.34	0.175	0.34
Gaski: Gambling	1.705	0.65	1.655	0.71	0.692	0.07	1.696	0.68	1.601	0.52	0.602	0.16

Note.^1^ANCOVA adjusted by sex and age and other gambling activity different to sports betting

^2^ANCOVA adjusted by sex and age and other gambling activity different to poker

SD: standard deviation

*Bold: significant comparison (*p*<0.05 level). ^†^Bold: effect size into the range mild-moderate to high-large

### Correlation and Predictive Analysis

Table 4 shows the partial correlation matrix (coefficients adjusted by sex and age). IGAS Involvement subscale and total scores were negatively associated (meaning higher involvement) with gambling severity level (SOGS total). Table 5 shows the results of the Poisson regression, which suggested that higher total scores on the CSCPK and with lower scores in the IGAS Involvement subscale (i.e., higher perceived impact of gambling advertising) predicted higher GD symptom severity level (SOGS total).

**Table 4:** Partial correlation matrix (adjusted by sex and age) (*N*=210)

	IGAS questionnaire				Bearden	Gaski-Etzel
	*Involvement*	*Awareness*	*Knowledge*	*Total*	*Total*	*Gambling*
Age of onset of GD	-0.017	-0.058	-0.093	-0.055	0.048	-0.067
Duration of GD	-0.097	-0.026	-0.102	-0.102	-0.058	0.052
SOGS total	**-0.316** ^**†**^	-0.141	-0.187	**-0.302** ^**†**^	0.132	-0.028
Debts due to GD	0.089	-0.063	0.064	0.061	0.115	-0.035
Illegal acts	-0.054	-0.018	-0.113	-0.075	-0.108	0.151

Note. GD: gambling disorder. SOGS: South Oaks Gambling Screen. ^†^Bold: effect size into the range mild-moderate to high-large

**Table 5:** Predictive model: Poisson’s regression adjusted by sex and age (*N*=210)

Criterion: SOGS total	B	SE	95% CI B		Wald	df	*p*
Sex (0: female, 1: male)	-0.046	0.081	-0.204	0.113	0.318	1	0.573
Gambling preference					2.390	2	
Non-strategic vs. mixed	-0.062	0.058	-0.175	0.051	1.158	1	0.282
Strategic vs. mixed	-0.084	0.058	-0.198	0.030	2.102	1	0.147
Gambling modality					2.342	2	
In-person vs. mixed	0.068	0.091	-0.111	0.247	0.547	1	0.460
Online vs. mixed	-0.015	0.103	-0.216	0.186	0.021	1	0.885
Social position (lower)	-0.025	0.021	-0.067	0.017	1.383	1	0.240
Age (years)	-0.002	0.002	-0.006	0.002	0.720	1	0.396
Duration of GD	0.006	0.004	-0.002	0.014	2.264	1	0.132
IGAS: Involvement	-0.086	0.029	-0.143	-0.029	8.850	1	0.003*
IGAS: Awareness	-0.007	0.025	-0.057	0.043	0.080	1	0.777
IGAS: Knowledge	-0.006	0.024	-0.053	0.042	0.055	1	0.815
Bearden: Total	0.037	0.018	0.001	0.073	4.069	1	0.044*
Gaski: Gambling	-0.016	0.032	-0.079	0.047	0.251	1	0.616

Note. GD: gambling disorder. SE: standard error. 95% CI: 95% confidence interval. *df*: degrees freedom

^*^Bold: significant parameter

## Discussion

The present study examined the perceived impact of gambling advertising among a sample of patients clinically diagnosed with gambling disorder (GD). The study results showed a mixed picture on the influence of gambling advertising. On the one hand, a statistically significant association was found between higher gambling advertising impact (Involvement subscale) and higher scores on gambling severity (supporting H_1_). On the other hand, gambling preferences (strategic versus non-strategic), and gambling modalities (in-person versus online) were not associated with gambling severity (therefore not supporting either H_2_ or H_3_).

The study found important associations between gambling severity and advertising. As aforementioned, H_1_ was supported because the perceived impact of gambling advertising was moderately correlated to gambling severity, and also because it was a factor in the prediction model. Three measures of perceived gambling advertising were predictors of gambling severity: the overall score, the involvement component of the perceived impact of gambling advertising (Hanss et al., 2015), and the persuasion knowledge scale (Bearden et al., 2001). Gamblers experiencing greater gambling problems reported being more aware of the gambling tactics, more cognizant about the strings attached to gambling promotions, more aware about the non-realistic worlds that advertising depicts, more able to recognize when a gambling operator was pressuring them to engage in gambling, and more capable of identifying offers ‘too good to be true’ (the items comprising the CSCPK). Simplistically, a higher score on persuasion knowledge should mean a higher ability to resist advertising impact but the present study showed the opposite, higher gambling symptomatology among those showing higher persuasion knowledge. It could be that gamblers experiencing problems might (in the long run) become more familiarized with gambling advertising and its mechanisms of persuasion, and by the time they reach psychological treatment they have developed a more critical understanding of advertising, they are more able to decode and neutralize their messages and are, in a sense, immune to them.

The finding of an association between the involvement component of the Impacts of Gambling Advertising Scale, and the total score of the scale, and gambling severity is relevant because it confirms the findings of previous studies suggesting such a relationship. Hitherto, the relationship between gambling severity and perceived impact of gambling advertising had been demonstrated using online panels (Hing et al., 2015, 2018), school surveys (Derevensky et al., 2010a; Noble et al., 2022), qualitatively, by means of focus groups or interviews (Lopez-Gonzalez et al., 2020), and overall by a recent umbrella review (McGrane et al., 2023). All these studies (excluding review papers) used non-clinical samples in which problem gambling was assessed using screening tools, which are prone to overestimate gambling severity and prevalence (Sturgis & Kuha, 2022).

The present paper uses a different kind of sample, exclusively recruited from a clinical setting, which entails a robust method of participant recruitment and identity verification. In addition, the dual method of GD diagnosis (questionnaire and in-person clinical interview) is less vulnerable to underestimation or overestimation. The results consolidate the evidence that gamblers experiencing problems report being more exposed to gambling advertising and their effects, particularly regarding their involvement. In this context, the involvement component covers items that are related to what happens in a gambler’s mind after viewing gambling advertising, including being more likely to gamble, becoming more interested in gambling in general, thinking more often about gambling, gambling in a riskier way, and thinking more positively about gambling. The involvement component is essential because it closely relates to the ability of gamblers experiencing problems to cease gambling, to stop thinking about it, or to gamble more safely.

As aforementioned, H_2_ and H_3_ were not supported by the study’s findings. Regarding H_2_, strategic gamblers did not perform differently from non-strategic gamblers in terms of perceived impact of gambling advertising. It was hypothesised that contemporary forms of strategic gambling are more attractive to young gamblers today, which translates into greater exposure to advertising and marketing stimuli for this subgroup. H_3_ followed the same logic as H_2_ because online gambling is supposed to engage younger gamblers and therefore this cohort would be more pervasively targeted by gambling advertisers. The results showed that these assumptions were mostly incorrect, although some differences were observed when the strategic subsample was divided into sports bettors and poker gamblers. Sports bettors and non-sports bettors did not show any differences, which was unexpected considering how sports betting advertising is not only prevalent, invasive, and difficult to ignore (Lopez-Gonzalez et al., 2017; Pitt et al., 2016), but also constitutes, in many jurisdictions, the largest proportion of marketing expenditure by gambling operators (Critchlow et al., 2022; Sproston et al., 2015). Similarly, there are data indicating that by 2018, online gambling advertising already represented 80% of the gambling advertising market (GambleAware, 2018). However, the results did not support neither H_2_ nor H_3_.

When refining the analysis, H_2_ was partially supported in the case of poker gamblers. Those who engaged in poker showed greater perceived impact of gambling advertising both for the involvement component and for the total IGAS score. This might be attributed to the fact that poker is considered by many as the most ‘strategic’ form of gambling, the one that involves the highest proportion of skill, knowledge, and strategy, and conversely, less dependence on luck. Gambling advertisements might present poker players with promotions and offers that can be interpreted as beneficial because their skills could be used for financial gains. The overreliance of poker players on their skilful abilities as opposed to other types of gamblers (Chrétien et al., 2017; Mathieu et al., 2018; Mihaylova et al., 2013) could make them more vulnerable to claims from gambling advertisers.

However, there is evidence suggesting poker players do not differ in terms of cognitions (e.g., cognitive flexibility) from other gamblers but they do in terms of some clinical characteristics (e.g., being male and of younger age) (Grant et al., 2012). An alternative interpretation of the results could be that poker players perceive themselves as more analytical and critical than other gamblers, and as a result find it less objectionable to acknowledge a theoretical impact of gambling advertising. The present study assessed the perceived (i.e., self-reported) impact of gambling advertising, which means it was inadequate in being able establish behaviour modification due to advertising stimuli.

### Limitations

The present study had some limitations. The data mostly relied on self-reported variables such as the perceived impact of gambling advertising and was therefore vulnerable to some biases. Recall bias might have caused some participants underestimate their exposure to gambling advertising. Social desirability bias might have worked both ways: on the one hand, overreporting the impact of gambling advertising to be perceived as critical and conscious individuals; and on the other hand, underreporting the impact to be perceived as an individual with the ability to resist commercial messages. Additionally, although the data covered a period of 18 months, it was a cross-sectional study that did not examine the persistence of the scores over a given period.

Another limitation is that the recruitment of participants was interrupted by the COVID-19 pandemic. Approximately two-thirds of the participants were recruited prior to the interruption and one-third after the interruption. In the present study, the effect of the pandemic on any of the studied variables was explored as it was not part of the research aims. However, the pandemic might have affected several aspects of the study, including gambling behaviour, higher exposure to gambling advertising due to staying longer hours at home during the confinement, and fluctuations in the number of individuals seeking for help for GD.

The sample descriptors vary from some previously collected samples in two meaningful ways. The treatment-seeking participants in the present study had a mean age of 39.4 years, which is slightly higher than the mean age reported in similar studies using online panels (36.7 years for a Spanish sample, and 35.7 years for an Australian sample in a previous comparative study) (Lopez-Gonzalez et al., 2020). Moreover, the proportion of females in the present study was very low (7.1%), which is comparatively less than this previous study (28% in Spain and 21% in Australia) In Spanish and Australian samples females showed greater gambling severity, which means that their underrepresentation here might have reduced the mean scores for gambling harm.

Finally, very few participants recruited by the end of the study might have been affected by a regulatory change in Spain. In November 2020, the Spanish gambling law severely reduced the amount of gambling advertising on television and radio, and imposing restrictions on the use of celebrities to sponsor gambling firms.

## Conclusion

The present study furthered the available empirical evidence indicating the existence of an association between gambling severity and gambling advertising. Those gamblers with GD experiencing more clinical symptoms were also the ones reporting higher perceived impact of gambling advertising. This finding is not novel per se, as has been previously implied by online panel studies, studies using registered users from gambling sites, and qualitative studies. However, to the best of our knowledge, this is one of the first empirical studies in which the association between gambling advertising and severity holds if studied among GD patients diagnosed in a clinical setting, free from the overestimations of gambling severity suspected from other recruitment procedures.

In many western countries, there is an increasing regulatory pressure on gambling operators to apply severe restrictions to what they can say in their commercial communication. The present study supports such measures by demonstrating that gambling advertising disproportionately affects those experiencing more gambling harm, with poker players with greater gambling severity being a particular group of interest for consumer protection regulations.

## Electronic supplementary material

Below is the link to the electronic supplementary material.

Supplementary Material 1

## Data Availability

A supplementary table is available for this study.

## Code Availability

Authors might share the code upon reasonable request.
