# Supplementary material for: Perceived Impact of Gambling Advertising can Predict Gambling Severity among Patients with Gambling Disorder
Source: J Gambl Stud. 2024 Jul 30;40(4):1787–803. doi: 10.1007/s10899-024-10342-2 (PMC11557654; doi:10.1007/s10899-024-10342-2)
Supplement: Supplementary file 1 — Supplementary Material 1 [file 10899_2024_10342_MOESM1_ESM.docx]

***Table S1. (supplementary)*** Descriptive data of the sample (N=210)

|  | *n* | *%* |  | *Age-onset-duration* | *Mean* | *SD* |
| --- | --- | --- | --- | --- | --- | --- |
| Sex Female | 15 | 7.1% |  | Age (yrs) | 39.40 | 13.30 |
| Male | 195 | 92.9% |  | Onset GD (yrs) | 28.87 | 11.91 |
| Marital status Single | 113 | 53.8% |  | Duration GD (yrs) | 5.21 | 5.65 |
| Married | 67 | 31.9% |  | *GD symptom severity* | *Mean* | *SD* |
| Divorced | 30 | 14.3% |  | DSM-5 criteria | 6.86 | 2.02 |
| Education Primary | 95 | 45.2% |  | SOGS total | 10.92 | 3.27 |
| Secondary | 88 | 41.9% |  | *GD impairing* | *n* | *%* |
| University | 27 | 12.9% |  | Debts due to GD | 41 | 19.5% |
| Employed No | 76 | 36.2% |  | Illegal acts due to GD | 55 | 26.2% |
| Yes | 134 | 63.8% |  | *Impact advertising* | *Mean* | *SD* |
| Social index High | 4 | 1.9% |  | IGAS: Involvement | 2.85 | 0.95 |
| Mean to high | 22 | 10.5% |  | IGAS: Awareness | 2.60 | 0.94 |
| Mean | 22 | 10.5% |  | IGAS: Knowledge | 2.61 | 1.09 |
| Mean to low | 86 | 41.0% |  | IGAS: Total | 2.74 | 0.80 |
| Low | 76 | 36.2% |  | Bearden et al.: Total | 2.99 | 1.18 |
| GD type Non-strategic | 81 | 38.6% |  | Gaski and Etzel: Gambling | 1.69 | 0.67 |
| Strategic | 76 | 36.2% |  |  |  |  |
| Mixed | 53 | 25.2% |  |  |  |  |
| GD modality In-person | 153 | 72.9% |  |  |  |  |
| Online | 44 | 21.0% |  |  |  |  |
| Mixed | 13 | 6.2% |  |  |  |  |

*Note.* GD: gambling disorder. SD: standard deviation.
